# Effect of IL‐17 on pulmonary artery smooth muscle cells and connective tissue disease‐associated pulmonary arterial hypertension

**DOI:** 10.1002/iid3.1243

**Published:** 2024-04-05

**Authors:** Tian‐Yan Shi, Xiao‐Hong Wen, Juan Meng, Yue‐Wu Lu

**Affiliations:** ^1^ Department of Rheumatology and Clinical Immunology, Beijing Chaoyang Hospital Capital Medical University Beijing China

**Keywords:** connective tissue disease‐associated pulmonary arterial hypertension (CTD‐PAH), ICAM, IL‐17, MAPK signal pathway, pulmonary artery smooth muscle cells (PASMCs)

## Abstract

**Objective:**

To explore the role of interleukin (IL)‐17 in connective tissue disease‐associated pulmonary arterial hypertension (CTD‐PAH) and to investigate its possible mechanism on pulmonary artery smooth muscle cells (PASMCs).

**Methods:**

Enzyme‐linked immunosorbent assay (ELISA) were used to compare levels of serum IL‐17 in patients with CTD‐PAH and healthy controls (HCs). After treatment for 3 months, the serum IL‐17 levels were tested in CTD‐PAH. ELISA and immunohistochemistry were used to compare levels of serum IL‐17 and numbers of pulmonary artery IL‐17^+^ cells, respectively, in a rat model of monocrotaline‐induced PAH and untreated rats. Proliferation, migration, and inflammatory factors expression of PASMCs were assessed after stimulation with different concentrations of IL‐17 for various time periods. Proteins in the mitogen‐activated protein kinase (MAPK) pathway were examined by western blot.

**Results:**

Levels of IL‐17 were upregulated in patients with CTD‐PAH compared to HCs. After 3 months of treatment, serum IL‐17 levels were downregulated with pulmonary artery pressure amelioration. Moreover, serum IL‐17 levels and numbers of IL‐17^+^ cells infiltrating lung arterioles were increased in PAH model rats. IL‐17 could dose‐ and time‐dependently promote proliferation and migration of PASMCs as well as time‐dependently induce IL‐6 and intercellular cell adhesion molecule‐1 (ICAM‐1) expression. The levels of MKK6 increased after IL‐17 treatment. Inhibition of MAPK decreased proliferation of PASMCs.

**Conclusion:**

Levels of IL‐17 may increase in CTD‐PAH, and IL‐17 promotes proliferation, migration, and secretion of IL‐6 and ICAM in PASMCs, respectively, which likely involves the p‐38 MAPK pathway.

## Graphical abstract

Levels of interleukin (IL)‐17 were upregulated in patients with connective tissue disease‐associated pulmonary arterial hypertension compared to healthy controls (HCs). After 3 months of treatment, serum IL‐17 levels were downregulated with pulmonary artery pressure amelioration. Moreover, serum IL‐17 levels and numbers of IL‐17^+^ cells infiltrating lung arterioles were increased in pulmonary arterial hypertension (PAH) model rats. IL‐17 could dose‐ and time‐dependently promote proliferation and migration of pulmonary artery smooth muscle cells as well as time‐dependently induce IL‐6 and intercellular cell adhesion molecule‐1 expression. The levels of MKK6 increased after IL‐17 treatment.

## 1 INTRODUCTION

Connective tissue disease‐associated pulmonary arterial hypertension (CTD‐PAH) is a disorder that has a high mortality rate.^1^ PAH is primarily characterized by increased pulmonary arterial resistance that leads to remodeling and thickening of pulmonary artery walls and eventually right heart failure.^2^ Recent accumulating evidence demonstrated that inflammation is a critical contributor to CTD‐PAH pathogenesis.^3^, ^4^ Inflammatory cells infiltrate the plexiform lesions both in patients with PAH and in animal models of PAH.^5^ Thus, pro‐inflammatory cytokines and chemokines produced by inflammatory cells may be responsible for the hyperproliferation of pulmonary artery smooth muscle cells (PASMCs).

Interleukin‐17 (IL‐17) is a vital pro‐inflammatory cytokine produced by Th17 cells and other cell types that binds to heteromeric receptor complexes comprising IL‐17RA and IL‐17RC subunits to activate signaling pathways such as nuclear factor‐kappa B (NF‐κB) or mitogen‐activated protein kinase (MAPK).^6^

A Th17/Treg cells imbalance was previously shown to be related to disease severity and prognosis in patients with CTD‐PAH, which suggests a role of IL‐17 for the development of PAH.^7^ IL‐17 activation may also be associated with vascular adventitial remodeling.^8^ However, the exact molecular mechanisms associated with IL‐17‐mediated CTD‐PAH and the interaction between IL‐17 and PASMCs remain elusive. Interpreting the crosstalk between IL‐17 and PASMCs is crucial to better understand the pathogenesis of CTD‐PAH and to provide evidence for anti‐IL‐17 as a potential therapeutic strategy.

In this study, we examined the level of serum IL‐17 in patients with CTD‐PAH and healthy controls (HCs). Serum IL‐17 levels were compared after treatment. We also analyzed the numbers of IL‐17‐positive cells in pulmonary artery wall samples and serum IL‐17 in rat models of CTD‐PAH. Finally, we examined the effect of IL‐17 on PASMCs to reveal probable mechanisms by which IL‐17 is involved in the development of CTD‐PAH.

## 2 MATERIALS AND METHODS

### 2.1 Patients and samples

The research was conducted in accordance with the Declaration of Helsinki and approved by an ethics committee in Beijing Chaoyang Hospital (AEEI‐2018‐068). A total of 30 patients with CTD‐PAH and 30 age‐ and sex‐matched HCs were enrolled in this study. Different CTDs were diagnosed according to the classification diagnostic criteria for each.^9^, ^10^, ^11^, ^12^, ^13^ In this present study, a diagnosis of PAH required a resting mean pulmonary artery pressure (mPAP) of ≥25 mmHg and a normal pulmonary capillary wedge pressure of ≤15 mmHg with right heart catheterization (RHC) (*n* = 20)^14^ or the calculation of systolic pulmonary artery pressure ≥50 mmHg by echocardiography (*n* = 30). As a control group, 30 subjects without CTDs, and other systemic and risk factors for cardiopulmonary diseases were included. PAH patients with other underlying etiology were excluded.^14^ Serum samples were collected from patients and HCs and stored at −80°C for enzyme‐linked immunosorbent assay (ELISA) analysis. The basic characteristics of the research subjects are shown in Table 1. Written informed consent was obtained from each study participant.

**Table 1:** Clinical characteristics of study participants.

	CTD‐PAH patients (*n* = 30)	Healthy controls (*n* = 30)	*p* Value
Age (year)	57.5 ± 10.5	58.9 ± 5.1	.51
Sex (male/female)	5/25	5/25	1.00
ESR (mm/h)	25.87 ± 21.76	8.43 ± 5.17	.0001
CRP (mg/dL)	1.2 (0.11, 6.70)	0.34 (0.10, 0.74)	.0002
IgG (mg/dL)	1725 ± 756	1239 ± 365	.005
IgA (mg/dL)	313 ± 135	293 ± 145	.59
IgM (mg/dL)	149 ± 115	103 ± 41	.06
C3 (mg/dL)	76.26 ± 22.74	107.20 ± 25.04	.0001
C4 (mg/dL)	14.42 ± 7.17	26.80 ± 8.93	.0001
eSPAP^a^ (mmHg; Echo)	80.1 ± 12.2		
eSPAP^b^ (mmHg; Echo)	64.2 ± 21.4		
eSPAP (mmHg; RHC)	66.5 ± 19.7 (*n* = 20)		
Diseases‐associated PAH	SSc (*n* = 14)		
	MCTD (*n* = 7)		
	pSS (*n* = 2)		
	SLE (*n* = 5)		
	DM (*n* = 1)		
	BS (*n* = 1)		
Treatment	GC + MMF + Ambrisentan (*n* = 6)		
	GC + CTX + Ambrisentan (*n* = 4)		
	GC + MMF (*n* = 3)		
	GC + CTX (*n* = 4)		
	CTX (*n* = 2)		
	MMF (*n* = 1)		
	CTX + Ambrisentan (*n* = 3)		
	MMF + Bosentan (*n* = 2)		
	MMF + Ambrisentan (*n* = 3)		
	CTX + Bosentan (*n* = 1)		
	MMF + Sildenafil (*n* = 1)		

Abbreviations: BS: Behcet's syndrome; CRP, C‐reactive protein; CTX, cyclophosphamide; DM, dermatomyositis; eSPAP, estimated systolic pulmonary artery pressure; ESR, erythrocyte sedimentation rate; GC, glucocorticoid; IgA, immunoglobulin A; IgG, immunoglobulin G; IgM, immunoglobulin M; MCTD, mixed connective tissue disease; MMF, mycophenolate mofetil; PAH, pulmonary arterial hypertension; pSS, primary Sjögren's syndrome; RHC, right heart catheterization; SLE, systemic lupus erythematosus; SSc, systemic sclerosis.

^a^ eSPAP at baseline.

^b^ eSPAP after treatment.

### 2.2 Animal models and samples

Monocrotaline (MCT; Sigma‐Aldrich) was dissolved in phosphate‐buffered saline. The pH was adjusted to 7.4 with HCl, and the volume was adjusted to achieve a final concentration of 20 mg/mL. Adult female Sprague‐Dawley rats (200–250 g body weight) were randomly divided into two groups and acclimatized for 5 days before receiving a single subcutaneous injection of either 60 mg/kg MCT or the same volume of saline (0.9% NaCl). After 21 days, all rats were euthanized for evaluation. Lung specimens were sectioned and fixed in buffered 10% formalin for 24 h at room temperature. All sections were embedded in paraffin and consecutive 4‐µm‐thick sections of the samples were stained with hematoxylin‐eosin.

### 2.3 Culture of rat PASMCs

PASMCs were trypsinized and seeded in plates in Dulbecco's modified Eagle's medium (GIBCO) supplemented with 10% fetal bovine serum (FBS), 100 U/mL penicillin (GIBCO), and 100 g/mL streptomycin (GIBCO). PASMCs were incubated at 37°C in humidified air with 5% CO_2_ and cultured in different plates according to the corresponding experiments. Characterization of PASMCs was performed by immunocytochemical staining with anti‐α‐smooth muscle actin (Sigma‐Aldrich).

### 2.4 Cell proliferation assay

A cell counting kit (CCK) 8 assay was used to identify cell proliferation based on the manufacturers' instructions. PASMCs (1 × 10^4^ cells/100 µL) were cultured overnight in 96‐well plates before stimulation with 0, 1, 5, or 10 ng/mL IL‐17 (Peprotech) for 24, 48, and 72 h, respectively. After the indicated time, 10 µL CCK 8 (Dojindo) solution was added to the wells and incubated for an additional 2 h. The optical density value of absorbance was measured at 450 nm. All experiments were performed in quadruplicate.

### 2.5 Cell migration assay

PASMC suspensions (100 µL of 1 × 10^5^ cells) were seeded in the upper chambers of a Transwell culture plate (Corning) in serum‐free medium in the presence of 0, 1, 5, or 10 ng/mL IL‐17. Then, 600 μL of medium containing 15% FBS was added to the bottom chamber. After incubation at 37°C and 5% CO_2_ for 24, 48, and 72 h, respectively, the cells were fixed with methanol and stained with crystal violet. The cells on the top surface of the membrane were gently wiped off with wet cotton swabs, and cells on the lower surface were examined. The average number of migrated cells was quantified by counting the number of cells in six randomly selected fields.

### 2.6 ELISA

PASMCs were cultured in 48‐well plates at 1 × 10^5^ cells/well in a total volume of 500 µL in the presence of 0, 1, 5, or 10 ng/mL IL‐17 at 37°C in humidified air with 5% CO_2_ for 24, 48, and 72 h, respectively. The culture supernatants were collected and stored for measurement of IL‐6 (eBioscience), IL‐21 (eBioscience), IL‐23 (eBioscience), and intercellular cell adhesion molecule‐1 (ICAM‐1) (eBioscience) with corresponding ELISA kits. The concentrations of IL‐17 in human and rat serum samples were measured using ELISA kits (eBioscience) according to the manufacturer's instructions.

### 2.7 Immunohistochemical analysis

Before immunohistochemical staining, the tissue sections were deparaffinized and rehydrated with water. Endogenous peroxidase was blocked with 3% H_2_O_2_ for 20 min at room temperature. The slides were treated with a 0.1 M citrate buffer in an 800 W microwave oven for 15 min for antigen retrieval, and then rinsed in distilled water and washed with phosphate‐buffered saline for 5 min. For immunohistochemical staining of IL‐17, 5% normal horse serum was used to suppress nonspecific protein binding, and 1 mg/mL anti‐rat‐IL17 antibody (1:100, ab214588; Abcam) was applied to the tissue sections and incubated overnight at 48°C. The slides were washed and incubated with enzyme‐conjugated secondary antibody for 1 h at room temperature. Color was developed with diaminobenzidine tetrahydrochloride.

### 2.8 Western blot analysis

PASMCs were lysed in radio‐Immunoprecipitation assay (RIPA) buffer containing phosphatase and protease inhibitors (ST505; Beyotime) on ice and centrifuged at 12,000 rpm for 20 min at 4°C. Protein concentrations were determined using a BCA assay (Beyotime) and equal amounts of protein (40 μg each lane) were subjected to SDS‐PAGE and then transferred onto polyvinylidene fluoride membranes (Millipore). Membranes were blocked with 5% fat‐free milk, and incubated overnight at 4°C with primary antibodies including anti‐MKK3 (AF6327; Affinity Biosciences), anti‐MKK6 (AF7820; Affinity Biosciences), anti‐P‐38 (AF6456; Affinity Biosciences), and anti‐glyceraldehyde‐3‐phosphate dehydrogenase (GAPDH) (ab181602; Abcam). After washing with PBST five times for 5 min each, the membranes were incubated with secondary antibodies for 1 h at room temperature. Target bands were quantified using a Tanon‐5200 Image Analyzer.

### 2.9 Quantitative reverse transcription polymerase chain reaction

Total RNA from PASMCs was extracted using TRIzol reagent (Invitrogen) according to the manufacturer's instructions, then reversely transcribed into complementary DNA (cDNA) using a Prime‐Script RT reagent kit (RR047A; Takara). cDNA was amplified with SYBR Premix Ex Taq™ II (RR820A; Takara). Using GAPDH as an internal control, expression of target genes was calculated by the relative quantitative method ($2‐∆∆C_{t}$ method). Experiments were conducted in triplicate independently. Primers sequences are listed in Table 2.

**Table 2:** Primer sequence for qRT‐PCR.

Gene	Primers (5′–3′)
IL‐6	F: TCC TAC CCC AAC TTC CAA TGC TC
	R: TTG GAT GGT CTT GGT CCT TAG CC
ICAM‐1	F: AGG TAT CCA TCC ATC CCA CA
	R: GCC ACA GTT CTC AAA GCA CA
GAPDH	F: GCT ATG AGC CCT TCC ACG ATG C
	R: GAA TCT ACT GCG TCT TCA CC

Abbreviations: GAPDH, glyceraldehyde‐3‐phosphate dehydrogenase; IL, interleukin; qRT‐PCR, quantitative reverse transcription polymerase chain reaction.

### 2.10 Statistical analysis

SPSS 17.0 software package (SPSS Inc.) was used for statistical analysis. Data were presented as the median (range) or mean ± standard deviation in intergroup comparisons. Student's t‐test or a nonparametric Mann–Whitney *U* test were used to determine differences between two groups, whereas one‐way analysis of variance was used to compare differences among multiple groups. Repeated measures analysis of variance was used to evaluate the continuous data. Statistical significance was defined as *p* <  .05.

## 3 RESULTS

### 3.1 Elevated serum IL‐17 decreased after treatment in patients with CTD‐PAH

**Figure 1:**
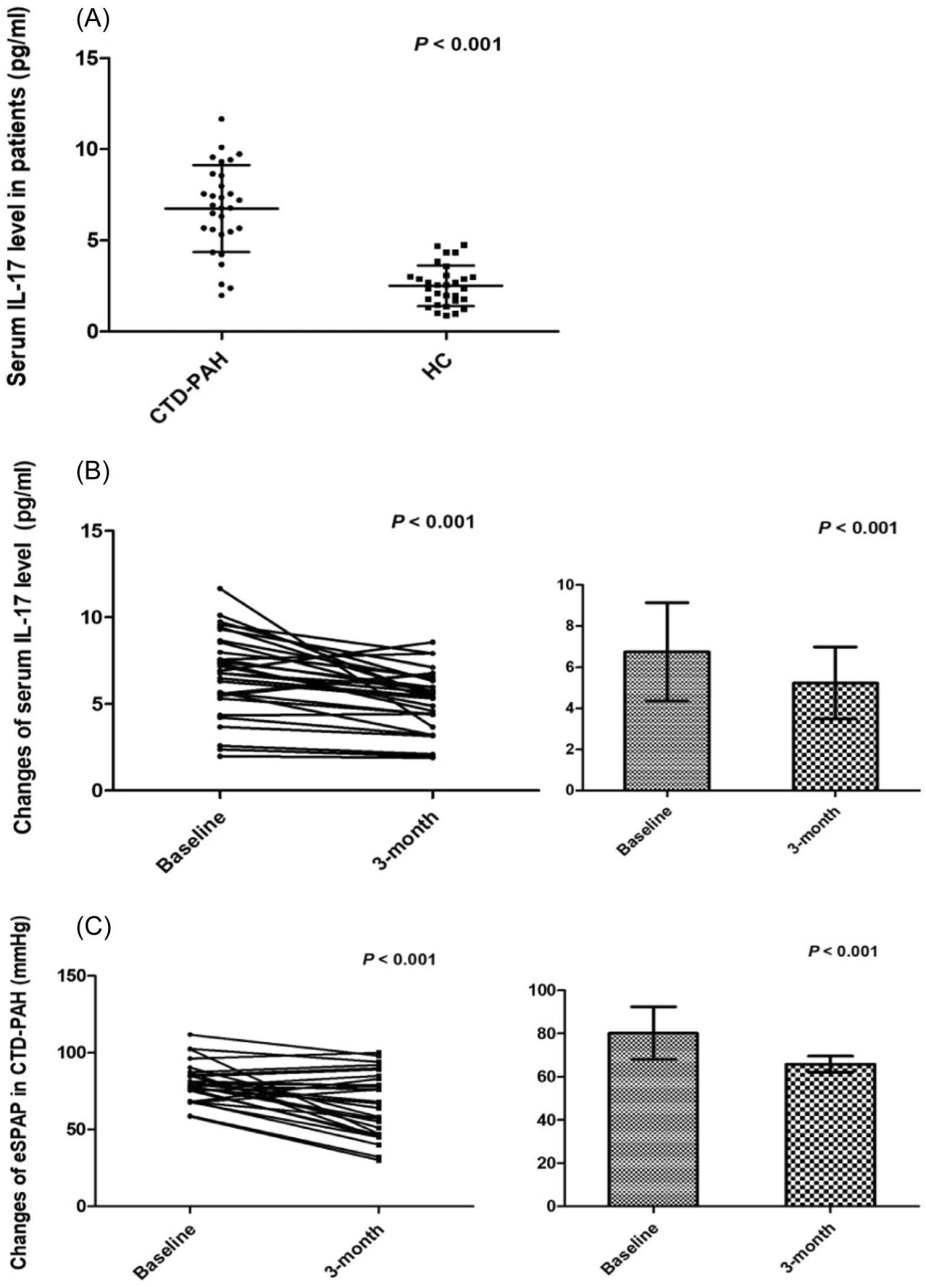
(A) Serum interleukin (IL)‐17 expression was elevated in patients with connective tissue disease‐associated pulmonary arterial hypertension (CTD‐PAH) compared to healthy controls (*n* = 30 each group) as measured by enzyme‐linked immunosorbent assay (6.75 ± 2.39 vs. 2.51 ± 1.11 pg/mL; *p* < .001); (B) serum IL‐17 expression changed in patients with CTD‐PAH (*n* = 30) after treatment (5.23 ± 1.75 vs. 6.75 ± 2.39 pg/mL; *p* < .001); (C) eSPAP was decreased in patients with CTD‐PAH (*n* = 30) as measured by ultrasound cardiogram after treatment (64.2 ± 21.4 vs. 80.1 ± 12.2 mmHg; *p* < .001).

A total of 30 patients with CTD‐PAH and 30 HCs were included. The serum IL‐17 was elevated in patients with CTD‐PAH compared with HCs (6.75 ± 2.39 and 2.51 ± 1.11 pg/mL, respectively; *p* < .001; Figure 1A). In addition, serum IL‐17 levels were decreased in patients after treatment, which was accompanied with pulmonary artery pressure amelioration (5.23 ± 1.75 and 6.75 ± 2.39 pg/mL, respectively; *p* < .001; Figure 1B).

### 3.2 Increased serum IL‐17 and infiltration of IL‐17 expressing cells in pulmonary artery walls in a rat model of PAH

**Figure 2:**
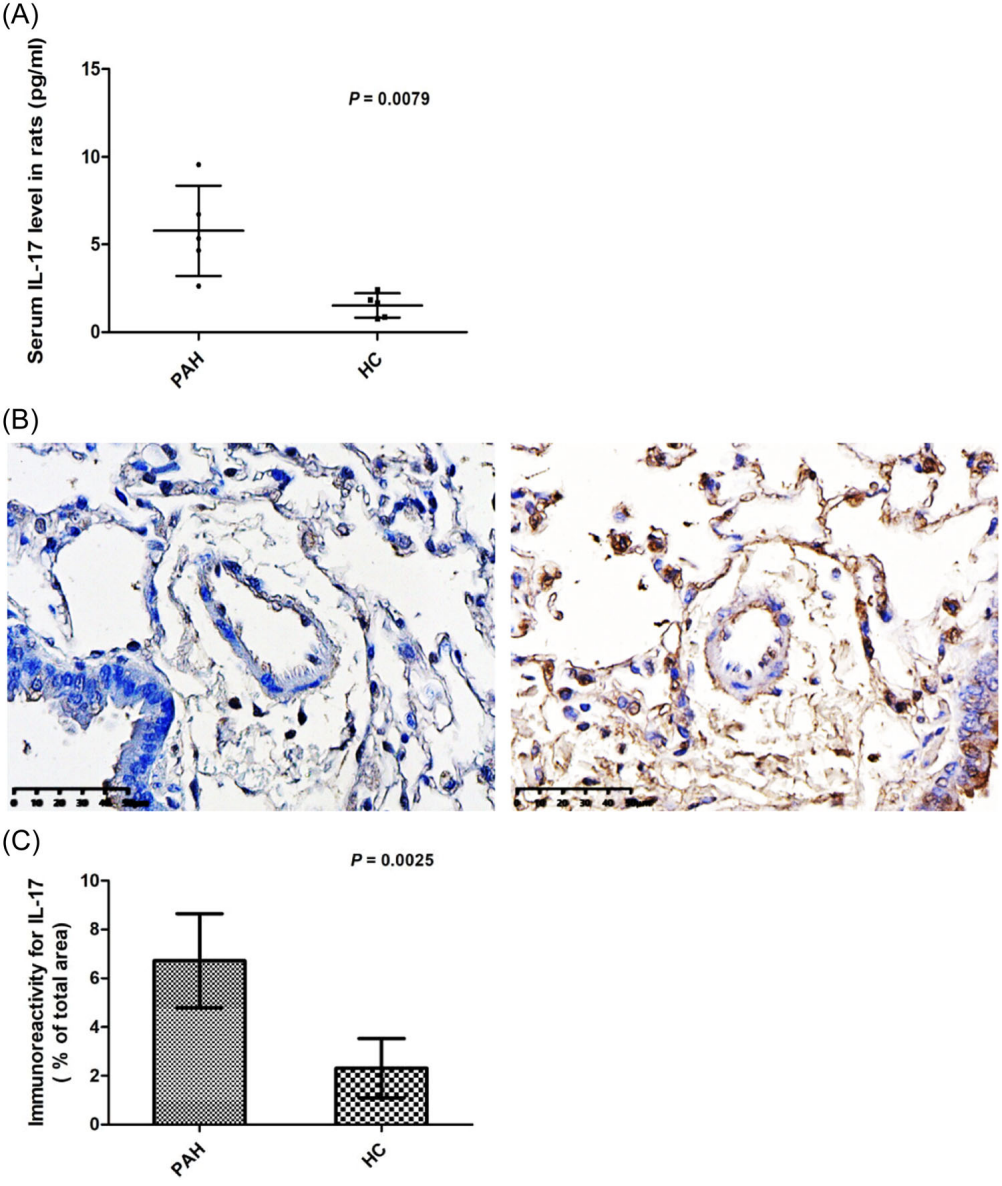
(A) Serum interleukin (IL)‐17 levels were upregulated in pulmonary arterial hypertension (PAH) rats compared to normal rats (*n* = 5 each group) (5.78 ± 2.58 vs. 1.58 ± 0.69 pg/mL; *p* = .0079); (B) immunohistochemical analysis shows that the number of IL‐17‐expressing cells was significantly increased in pulmonary artery walls from PAH model rats; (C) immunoreactivity for IL‐17 in pulmonary artery walls from PAH rats was higher compared to normal rats (*n* =  5 each group) (6.72 ± 1.93 vs. 2.31 ± 1.21; *p* = .0025). HC, healthy control.

Compared to normal controls (*n* = 5), ELISA showed that serum levels of IL‐17 were upregulated in a rat model of PAH (*n* = 5) (5.78 ± 2.58 and 1.58 ± 0.69 pg/mL, respectively; *p* = .0079; Figure 2A). In terms of disease characteristics, organ infiltration of IL‐17 is known to be more central than serum levels. Immunohistochemistry showed the number of IL‐17 expressing cells was significantly increased in pulmonary artery walls from PAH model rats compared to normal rats (6.72 ± 1.93 and 2.31 ± 1.21, respectively; *p* = .0025; Figure 2B,C). Moreover, the number of IL‐17‐positive inflammatory cells was increased in lung sections from PAH rats.

### 3.3 IL‐17 dose‐ and time‐dependently promoted proliferation and migration of PASMCs

**Figure 3:**
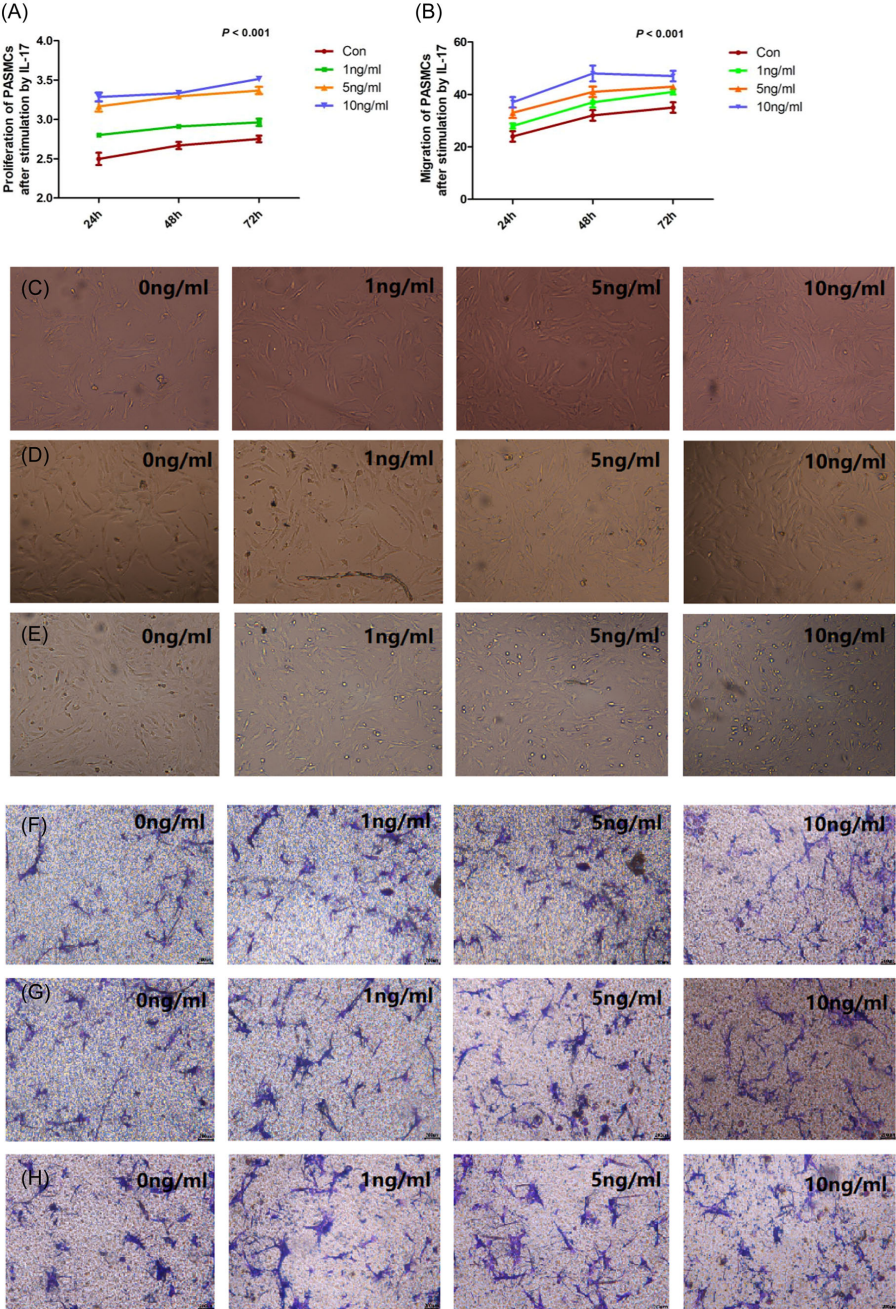
Pulmonary artery smooth muscle cells (PASMCs) (A) proliferation and (B) migration were dose‐ and time‐dependently enhanced by interleukin (IL)‐17; (C), (D), and (E) proliferation of PASMCs at 24 h (2.50 ± 0.05, 2.80 ± 0.01, 3.17 ± 0.07, 3.26 ± 0.06; *p* < .001), 48 h (2.67 ± 0.05, 2.91 ± 0.01, 3.29 ± 0.02, 3.33 ± 0.03; *p* < .001), and 72 h (2.75 ± 0.04, 2.96 ± 0.05, 3.37 ± 0.05, 3.52 ± 0.03; *p* < .001) after stimulation with IL‐17 at different concentrations, respectively; (F–H) migration of PASMCs at 24 h (24 ± 2, 28 ± 1, 33 ± 2, 37 ± 2; *p* < .001), 48 h (32 ± 2, 37 ± 2, 41 ± 2, 48 ± 3; *p* < .001), and 72 h (35 ± 2, 41 ± 1, 43 ± 1, 47 ± 2; *p* < .001) after stimulation with IL‐17 at different concentrations, respectively (*n* = 5 each group).

PASMCs can be activated by many factors that sequentially contribute to the development of PAH. We cultured rat PASMCs with 0, 1, 5, or 10 ng/mL IL‐17 for different time periods. Both IL‐17 concentration and culture time could affect PAMSCs proliferation (*p* < .001; Figure 3A) and migration (*p* < .001; Figure 3B).

### 3.4 IL‐17 dose‐dependently enhanced IL‐6 and ICAM‐1 production in PASMCs

**Figure 4:**
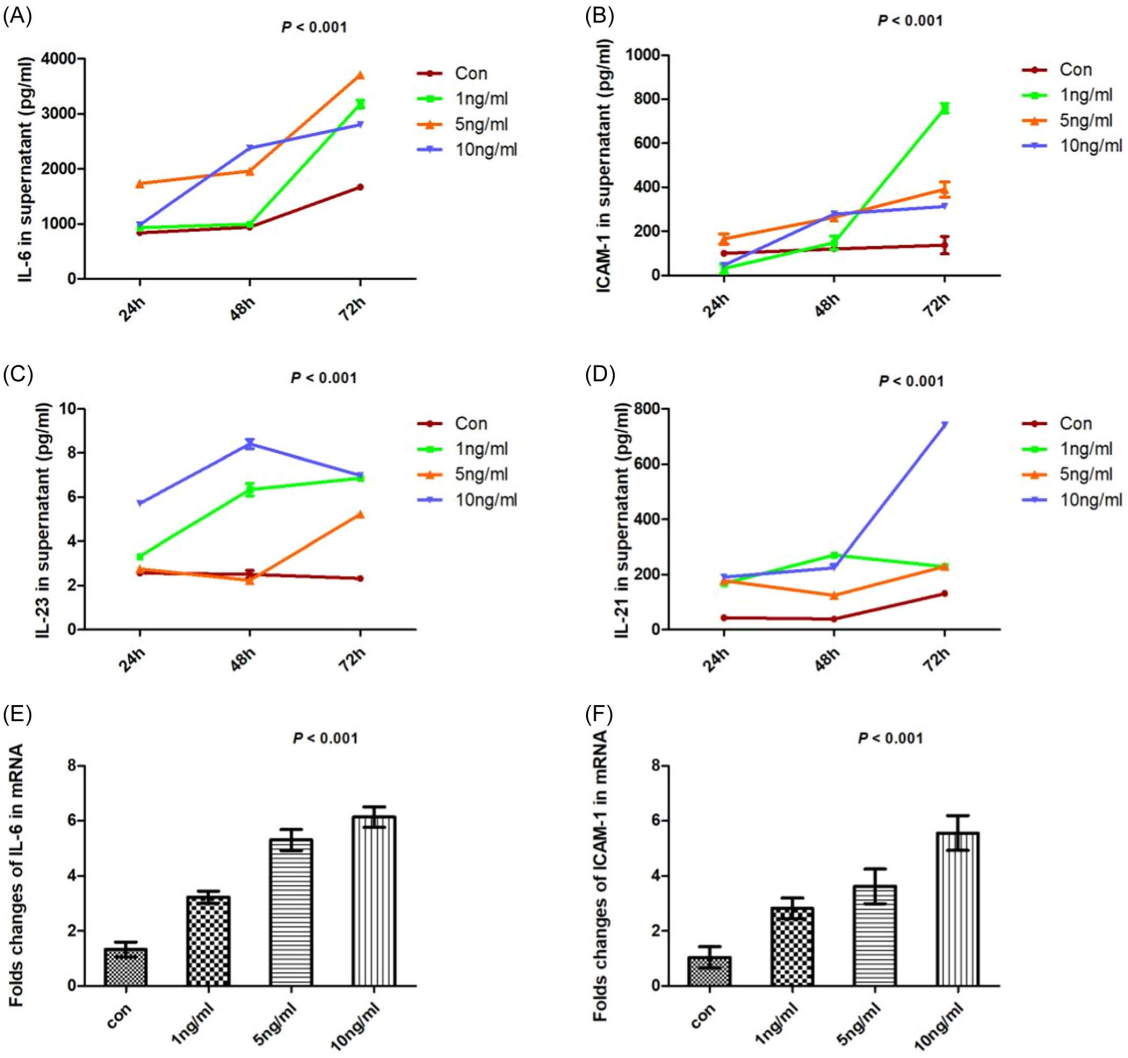
(A) Stimulation with different concentrations of interleukin (IL)‐17 was associated with elevated levels of IL‐6 at 24 h (842.9 ± 10.2, 935.5 ± 37.4, 1734.1 ± 75.2, 983.0 ± 16.0 pg/mL; *p* < .001), 48 h (997.9 ± 3.7, 935.5 ± 37.4, 1961.3 ± 13.0, 2378.0 ± 12.9 pg/mL; *p* < .001), and 72 h (1673.1 ± 41.0, 3178.6 ± 71.3, 3708.0 ± 10.4, 2801.7 ± 41.6 pg/mL; *p* < .001), respectively, particularly at 48 h; (B) stimulation with different concentrations of IL‐17 was associated with elevated levels of ICAM‐1 at 24 h (99.9 ± 12.7, 30.2 ± 21.9, 165.2 ± 23.2, 45.5 ± 9.9 pg/mL; *p* < .001), 48 h (120.5 ± 8.2, 148.5 ± 31.5, 262.5 ± 10.9, 277.8 ± 10.8 pg/mL; *p* < .001), and 72 h (137.1 ± 39.9, 759.0 ± 21.3, 390.0 ± 34.9, 313.1 ± 10.6 pg/mL; *p* < .001), respectively, particularly at 48 h; (C) stimulation with IL‐17 affected IL‐23 production at 24 h (2.57 ± 0.03, 3.33 ± 0.11, 2.75 ± 0.05, 5.72 ± 0.13 pg/mL; *p* < .001), 48 h (2.51 ± 0.17, 6.35 ± 0.29, 2.24 ± 0.09, 8.41 ± 0.21 pg/mL; *p* < .001), and 72 h (2.32 ± 0.03, 6.86 ± 0.08, 5.24 ± 0.18, 6.99 ± 0.10 pg/mL; *p* < .001), respectively, but there was no defined trend; (D) stimulation with IL‐17 affected IL‐21 production at 24 h (43.9 ± 1.3, 167.1 ± 1.14, 178.5 ± 1.16, 190.9 ± 3.9 pg/mL; *p* < .001), 48 h (38.4 ± 5.2, 271.6 ± 0.4, 124.5 ± 0.7 L, 224.7 ± 11.6 pg/mL; *p* < .001), and 72 h (131.1 ± 4.4, 228.8 ± 9.2, 230.0 ± 2.5, 741.1 ± 6.6 pg/mL; *p* < .001), respectively, but there was no defined trend either; (E) stimulation with different concentrations of IL‐17 elevated gene expression of IL‐6 at 48 h (1.3 ± 0.3, 3.2 ± 0.5, 5.3 ± 0.9, 6.1 ± 0.8; *p* < .001); (F) stimulation with different concentrations of IL‐17 elevated gene expression of ICAM‐1 at 48 h (1.0 ± 0.4, 2.8 ± 0.8, 3.6 ± 1.4, 5.5 ± 1.4; *p* < .001) (*n* = 5 each group).

We examined expression of IL‐6, ICAM‐1, IL‐21, and IL‐23 in supernatants of PASMCs cultured with various amounts of IL‐17 at different time points. The levels of IL‐6 increased at 24 h (842.9 ± 10.2, 935.5 ± 37.4, 1734.1 ± 75.2, and 983.0 ± 16.0 pg/mL, respectively; *p* < .001), 48 h (997.9 ± 3.7, 935.5 ± 37.4, 1961.3 ± 13.0, and 2378.0 ± 12.9 pg/mL, respectively; *p* < .001), and 72 h (1673.1 ± 41.0, 3178.6 ± 71.3, 3708.0 ± 10.4, and 2801.7 ± 41.6 pg/mL, respectively; *p* < .001; Figure 4A) after treatment with diverse IL‐17. The similar change were found in ICAM‐1 expression at 24 h (99.9 ± 12.7, 30.2 ± 21.9, 165.2 ± 23.2, and 45.5 ± 9.9 pg/mL, respectively; *p* < .001), 48 h (120.5 ± 8.2, 148.5 ± 31.5, 262.5 ± 10.9, and 277.8 ± 10.8 pg/mL, respectively; *p* < .001), and 72 h (137.1 ± 39.9, 759.0 ± 21.3, 390.0 ± 34.9, and 313.1 ± 10.6 pg/mL, respectively; *p* < .001; Figure 4B). At 48‐h time point in particular, IL‐17 dose‐dependently stimulated IL‐6 and ICAM‐1 secretion. Meanwhile, IL‐23 levels (24 h: 2.57 ± 0.03, 3.33 ± 0.11, 2.75 ± 0.05, and 5.72 ± 0.13 pg/mL; *p* < .001; 48 h: 2.51 ± 0.17, 6.35 ± 0.29, 2.24 ± 0.09, and 8.41 ± 0.21 pg/mL; *p* < .001; 72 h: 2.32 ± 0.03, 6.86 ± 0.08, 5.24 ± 0.18, and 6.99 ± 0.10 pg/mL; *p* < .001; Figure 4C) and IL‐21 levels (24 h: 43.9 ± 1.3, 167.1 ± 1.14, 178.5 ± 1.16, and 190.9 ± 3.9 pg/mL; *p* < .001; 48 h: 38.4 ± 5.2, 271.6 ± 0.4, 124.5 ± 0.7 L, and 224.7 ± 11.6 pg/mL; *p* < .001; 72 h: 131.1 ± 4.4, 228.8 ± 9.2, 230.0 ± 2.5, and 741.1 ± 6.6 pg/mL; *p* < .001; Figure 4D) differed significantly, but no obvious trends were observed. We also found that gene expression of IL‐6 (1.3 ± 0.3, 3.2 ± 0.5, 5.3 ± 0.9, and 6.1 ± 0.8, respectively; *p* < .001; Figure 4E) and ICAM‐1 (1.0 ± 0.4, 2.8 ± 0.8, 3.6 ± 1.4, and 5.5 ± 1.4, respectively; *p* < .001; Figure 4F) was induced by diverse concentrations of IL‐17 after 48 h of treatment.

### 3.5 MKK6 levels were elevated in PASMCs after IL‐17 stimulation

**Figure 5:**
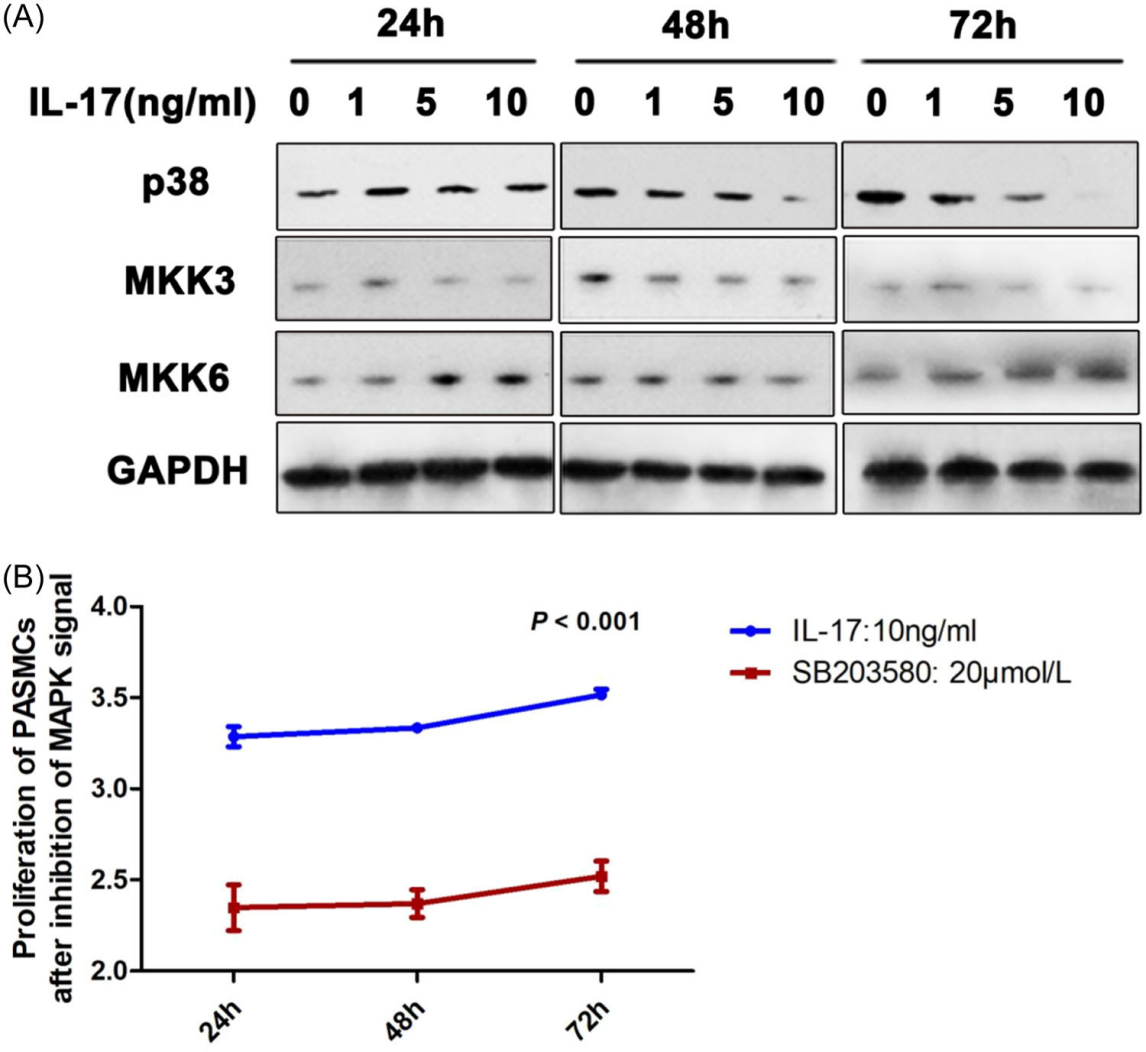
(A) Protein expression of mitogen‐activated protein kinase signal pathway components; (B) inhibition with a highly specific p38 inhibitor decreased proliferation of pulmonary artery smooth muscle cells (PASMCs) at 24 h (2.34 ± 0.13 vs. 3.29 ± 0.06; *p*＜.001), 48 h (2.37 ± 0.08 vs. 3.33 ± 0.03; *p*＜0.001) and 72 h (2.52 ± 0.08 vs. 3.52 ± 0.03; *p*＜.001), respectively (*n* = 5 each group). GAPDH, glyceraldehyde‐3‐phosphate dehydrogenase; IL, interleukin; MAPK, mitogen‐activated protein kinase.

Based on the above results indicating that IL‐17 plays a role in pulmonary vascular remodeling both in vitro and in vivo, we explored the mechanism associated with the effects of IL‐17. The levels of MKK6 were dose‐dependently upregulated in PAMSCs in the presence of IL‐17 both at 24 and 72 h in vitro as shown by western blot analysis (Figure 5A). However, the expression levels of the genes listed above were not statistically different. To further demonstrate the effects of MAPK on PAMSCs, cultures were treated with SB203580, a highly specific p38 inhibitor, at the concentration of 20 μmol/L.^15^ This resulted in decreased proliferation of PASMCs at 24 h (2.34 ± 0.13 and 3.29 ± 0.06; *p*＜.001), 48 h (2.37 ± 0.08 and 3.33 ± 0.03; *p*＜.001), and 72 h (2.52 ± 0.08 and 3.52 ± 0.03; *p*＜.001), respectively (Figure 5B).

## 4 DISCUSSION

PAH has a complex pathogenesis to which dysregulated immune conditions and abnormal migration and proliferation of PAMSCs are critical contributors.^16^ Inflammation is a hallmark characteristic of PAH.^16^, ^17^ In the present study, we demonstrated that: (1) levels of serum IL‐17 were upregulated in patients with CTD‐PAH compared to HCs; moreover, serum IL‐17 levels were downregulated after treatment concomitant with pulmonary artery pressure amelioration; (2) serum IL‐17 and infiltration of IL‐17^+^ cells in lung arterioles were increased in a rat model of PAH; (3) IL‐17 can dose‐ and time‐dependently promote PASMC proliferation and migration; (4) IL‐17 time‐dependently induced IL‐6 and ICAM‐1 expression in PASMCs and at 48 h the expression exhibited a dose‐dependent effect of IL‐17; and (5) the MAPK signal pathway may participate in PAH after stimulated by IL‐17.

Several lines of evidence suggest that inflammation is involved in the initiation and/or progression of PAH.^5^ First, altered immune cell populations and related cytokines and chemokines have been observed in blood from patients with PAH as well as in animal models of PAH.^18^ Several previous studies found that the level of serum cytokines correlated with pulmonary hemodynamics and clinical outcomes.^19^ Furthermore, multiple immune cells and inflammatory factors have been detected in perivascular spaces around target organs. The extensive distribution of the IL‐17 receptor complex allows IL‐17 to act on many cell types. IL‐17 is known to be involved in a variety of pathophysiological processes and multiple diseases such as systemic lupus erythematosus^20^ and primary biliary cirrhosis,^21^ as well as many lung diseases^22^ such as asthma, pneumonitis, and pulmonary fibrosis. However, whether IL‐17 has a direct pathogenic role or acts as a downstream factor in PAH is unclear.

Significant differences in the percentages and absolute counts of Th17 cells were found between patients with CTD‐PAH compared to those with CTD only.^7^ Furthermore, the percentage of Th17 cells was higher for patients with severe CTD‐PAH compared to those with mild to moderate disease.^7^ This result supported that the balance between Th17 and Treg cells influence the prognosis of CTD‐PAH.^7^ Harbaum et al.^23^ found that IL‐17 level was increased in some patients with idiopathic PAH. CD4^+^ T cells in PAH patients may express higher levels of IL‐17 after activation.^24^ Meanwhile, Wang et al.^25^ demonstrated that IL‐17^−/−^ mice were resistant to development of hypoxic pulmonary hypertension (HPH) and that serum IL‐17 levels were upregulated in patients with HPH. Treatment with an inhibitor of Th17 cell development can decrease blood pressure and remodeling responses to chronic hypoxia in a mouse model of PAH.^26^ Consistent with previous studies, we found increased serum IL‐17 in patients with CTD‐PAH, as well as increased infiltration of IL‐17^+^ cells in pulmonary arteries in a rat model of PAH. The decreased levels of IL‐17 was accompanied with alleviation of pulmonary artery pressure after treatment, which all support the role of IL‐17 in PAH pathogenesis.

The existence of crosstalk between vascular cells and inflammation that mediates the development of PAH is widely accepted. IL‐17 alone or in combination with other cytokines may affect all cell types that make up the three layers of the vascular wall.^27^ In PAH, PASMCs, which are the most crucial components of the pulmonary vascular wall, have altered sensitivity to inflammation and an enhanced capacity to secrete cytokines and chemokines. Synergistically with interferon (IFN)‐γ, IL‐17 appeared to induce vascular smooth muscle cells to promote production of cytokines and chemokines.^28^ Liu et al.^29^ reported that IL‐17 prompted endothelial nitric oxide synthase expression and nitric oxide production in human vascular endothelial cells in transplant vasculopathy. Wang et al.^25^ described that hypoxia upregulated IL‐17 expression that affected pulmonary vascular remodeling by modulating pulmonary arterial endothelial cell functions. In a study by Numasaki, IL‐17 was shown to stimulate the growth and production of proangiogenic factors of vascular endothelial cells.^30^ Acting together with IL‐6, IL‐17A‐induced monocytes adhere to vessels via increased ICAM‐1 expression.^31^ Moreover, IL‐17A administration could induce inward remodeling of small arteries in model mice through hypertrophy and phenotype changes of vascular smooth muscle cells.^32^ In adult patients with PAH, ICAM‐1 levels were increased and significantly correlated with mPAP.^33^ Data from our present study showed that IL‐17 significantly enhanced PASMCs proliferation and migration in a dose‐ and time‐dependent manner. When PASMCs were cultured with IL‐17, we found that the expression of IL‐6 and ICAM‐1 increased with increasing culture time. At the 48‐h time point, IL‐17 had dose‐dependent effects on IL‐6 and ICAM‐1 gene expressions and protein secretion.

IL‐17 activates several signaling pathways including the canonical NF‐κB, MEK‐ERK1/2, phosphatidylinositol 3‐kinases‐Akt, c‐Jun N‐terminal kinase β‐catenin, and p38‐MAPK pathways.^34^ However, the pathways mediated by IL‐17 in PAH are not well elucidated. In vivo, modulation of the balance of Th17/Treg cells by targeting of the RhoA‐ROCK pathway can induce pulmonary vascular remodeling in HPH rats.^35^ IL‐17A stimulated aortic endothelial cells^31^ and chemokines expression by lung microvascular endothelial cells^36^ via the p38‐MAPK pathway. Moreover, inhibition of p38‐MAPK decreased this IL‐17A‐mediated activation by ameliorating IL‐6 and ICAM‐1 expression.^31^ In mice, IL‐17 treatment increased systolic blood pressure, which was associated with decreased aortic NO‐dependent relaxation responses and increased RhoA expression.^37^ In the present study, we found that expression levels of MMK6 increased in PASMCs cultured with IL‐17. Inhibition of the MAPK signaling pathway decreased the proliferation of PASMCs.

The current study has some limitations. First, RHC cannot be performed on every patient in routine clinical practice. In our study, we identified 66.7% of patients with CTD‐PAH based on RHC. Second, pulmonary vascular specimens were not available to determine IL‐17 levels in tissues of patients with CTD‐PAH, which may show more definitive evidence for the role of IL‐17 in this disease. Third, the effects of inhibiting the MAPK signaling pathway were not assessed comprehensively.

In conclusion, our results demonstrate that IL‐17 may contribute to promoting proliferation, migration, and secretion of IL‐6 and ICAM by PASMCs and this effect may occur via the p‐38 MAPK pathway.

## AUTHOR CONTRIBUTIONS

**Tian‐Yan Shi**: Conceptualization; data curation; formal analysis; funding acquisition; investigation; methodology; project administration; writing—original draft; writing—review and editing. **Xiao‐Hong Wen**: Data curation; formal analysis; investigation; resources. **Juan Meng**: Data curation; resources. **Yue‐Wu Lu**: Formal analysis; supervision.

## CONFLICT OF INTEREST STATEMENT

The authors declare no conflict of interest.

## ETHICS STATEMENT

The study was approved by the Ethics Committee of Beijing Chaoyang Hospital. All procedures were performed in accordance with the Declaration of Helsinki. Signed informed consent was obtained from all participants.

## Data Availability

The data sets used and/or analyzed during the current study are available from the corresponding author upon reasonable request.
